# Notes of Medico-Legal Cases

**Published:** 1886-07

**Authors:** S. V. Clevenger

**Affiliations:** Consulting Physician to Michael Reese Hospital, etc., etc.


### DOMESTIC CORRESPONDENCE.

NOTES OF MEDICO-LEGAL CASES OF NERVOUS AND MENTAL DISEASE. *By* S. V. CLEVENGER, M. D., *Consulting Physician to Michael Reese Hospital, etc., etc.*

*To the Editors of the* CHICAGO MEDICAL JOURNAL AND EXAMINER:

GENTLEMEN.—I send you herewith notes of some of the medico-legal cases in which I was recently engaged as an expert, and which presented many points of general and special interest to physicians and lawyers. Although outlines only are given below, yet these may serve as indices to the cases which are recorded in full by the courts and attorneys, where use for details may be found.

PARANOIA.

*State vs. Otto Funk.*—This individual was well known as the book-thief and dynamiter. His life was that of the typical paranoiac. He studied at the Chicago University and filled his rooms with books stolen from the public library of the city, between one and two thousand volumes, it is said. He had a picture of a pretty nun on his wall before which he would spend hours. Finally he discovered a young lady who resembled the one in the picture, and for years annoyed her with his attentions. As she repelled him, he dug a trench in the rear of the University and at one end of it placed an ingeniously constructed trap for the purpose of abducting her.

I asked him, when he was in prison, what his intentions were with regard to her, and he stated that he would have indelibly marked her forehead with "Variety is the spice of life," and then let her go. But, said I, "You had many opportunities to do this in the college building without resorting to such troublesome methods as digging pit-falls." He replied, "I never thought of that."

Among the cart-loads of books recovered from Funk was a cake of dynamite. He had no very definite ideas as to what he intended doing with it though it must have had some connection with certain reformatory and socialistic schemes he entertained.

Like Guiteau he had written a book, though it had not been printed. The wording was sophomoric and egotistical. He was the central figure on every page. As revealed by his scribblings, he delighted in imagining himself wrongfully dispossessed of a title to nobility and an immense fortune. He repudiated his plebeian parents and their uneuphonious name, assuming Talcott as a cognomen. According to his narrative, he was learned, mysterious, handsome and irresistible in love. His erotism seems to have been platonic, however.

Spitzka describes such characters as having "bizarre rather than productive conceits, their reasoning as paradoxical rather than logical, and their argumentation tricky and shrewd rather than substantial. To the laity such subjects often appear to be brighter than the ordinary run of mankind, because commonly oddity is mistaken for brilliancy and unblushing pretense for merit." There was a marked occipital flattening, such as Morel noticed as possessed by some paranoiacs, with a not very noticeable asymmetry of the skull and tectocephalism.

He was committed to the Elgin Asylum, from which he escaped and managed to enter the Harvard Theological School, in Cambridge, Massachusetts. He stole large quantities of books from the library of this institution, was detected and committed suicide in prison.

EPILEPTIC INSANITY.

*State vs. Gardner.* An itinerant dentist had neglected and abused his wife in many ways. It was proven that she had purchased a revolver, and a week later had shot her husband and infant and attempted her own life. At the hospital she aroused from a stupor and asked to be allowed to return home, or that her husband might be summoned. She plainly had no recollection of the tragedy. At the request of the Woman's Club, of Chicago, and Attorney Westover, I examined into the case and testified that the act was consistent with the belief that Mrs. Gardner was suffering from larvated epileptic insanity. It transpired in the evidence that her father was an epileptic and that Mrs. G. had frequent attacks of facial spasms, "Jacksonian" epilepsy and *petit mal.* The deliberation with which she set about the slaughter had no weight against my theory, notwithstanding the ground held by some alienists, that absence of design is the main characteristic of furious acts of the epileptic insane.

Echeverria, in the *Journal of Mental Science*, London, April, 1885, concludes an examination of this question as follows:

"Sudden impulsive acts related to the psychical manifestations of epilepsy very often evince in their automatic execution a coherent planned purpose.

"Epileptics cannot be held responsible for any act of violence perpetrated during their unconscious automatism, which they have no power to control or capacity to judge."

The testimony was uniformly to the effect that she had been a devoted wife and that she was wholly unconscious at the time of the killing. Judge Moran, before whom the case was tried, was evidently convinced of the irresponsibility of Mrs. Gardner, and the jury unhesitatingly acquitted her.

SPINAL CONCUSSION.

*Holland vs. C. & E. I. R. R. Co.* February 16, 1883, Isaac W. Holland, a conductor on a suburban passenger train of the C. & R. I. R. R., was thrown forward by a collision, against the front of his car, rebounding backward, striking the last dorsal vertebræ upon the solid wooden top part of a seat, thence whirling to the floor. When he arose he felt sharp tingling sensations in his back, neck, head and extremities. He walked about, however, at the time, and assisted in getting his train into shape. The force of the collision may be judged from both locomotives being wrecked. At this date he weighed 197 pounds, and two years later only 102 pounds.

March 25th, 1883, the pain in his back became so severe as to necessitate his giving up work, but he rallied for a few days, struggling manfully against his fate, with slight remissions, until December 9th, 1883, when he became bedridden, in which condition he has remained ever since.

In December, 1884, at the request of plaintiff's attorneys, Messrs. Kretzinger, I visited and examined Mr. Holland, and found complete harmony between the subjective symptoms and the objective signs of his disorder. He was in a darkened room, his pupils were dilated and he suffered from photophobia, constant pain in his back; noises made his head ache. He had not had a good night's sleep for a year. His appetite was miserable. In his arms and legs, as well as rest of body, temperature was subnormal, and formication and numbness were constant. Pain began in the dorsal region and shot up to his head. Muscular motions were feeble, emaciation was extreme. He was unable to move himself in bed without assistance. Genital power and sexual desire were extinguished. He had had several epileptiform convulsions in the past year.

Tested with the æsthesiometer, there was a diminished ability to determine or locate points touched. With hyperæsthesia there was an increased sensory response to both the galvanic and faradic currents, indicative of irritability, but no reactions of degeneration.

Dr. Bettman, the oculist, at my request, made an ophthalmoscopic examination of the retinæ, without atropine, as the pupils were sufficiently dilated, and found anæmic discs. Fibrillary tremors of the muscles of arms and legs were noticeable. The tendon reflexes were greatly exaggerated, the stroke below the patellæ caused intense pain in the back. Limb coördination was decidedly impaired, he could not touch his nose tip with his eyes closed.

Plaintiff brought suit against the Eastern Illinois R. R. Co., as the one responsible for the injuries he had sustained, claiming $40,000 damages for total disability. The trial in Judge Gary's court, May, 1885, lasted eight days.

Simulation was the main claim for the defence and Page's little work on "Injuries to the Spine" was used by defendant's attorneys to establish the grounds taken.

Plaintiff's attorney made excellent use of Ericksen, Ross, Gowers, Hartshorne, Pavy, and numerous other medico-legal, neurological, surgical and general medical works, to show the consistency of Holland's condition with the usual phenomena of spinal concussion and to defeat the claim of the rail road that he was undergoing voluntary starvation.

The jury awarded plaintiff $25,000 damages and the case went to the appellate court, appellants claiming the amount to be excessive. In March, 1886, the award of the lower court was approved and the judgment sustained.

INSANITY A SYMPTOM OF BODILY DISEASE.

*Crandall vs. Accident Insurance Company of North America.*—An action was brought in Judge Wylie's United States District Court, to recover on a $10,000 policy. Mr. Thomas Bates, attorney for defendant, held that Mr. Crandall had committed suicide while insane and that provision had been made in the policy against death or injury arising from diseased conditions of the holder. Mr. Bates engaged me to testify as to the relationship existing between insanity and bodily disease.

I held that insanity was a disease of the mind, just as neuralgia was a disease of the nerve function, but it was only a *symptom* of a bodily disease. The distinction being that the mind as an effect of normal bodily workings could be deranged only through some improper action of bodily organs. It is inconceivable that the time-keeping of a clock should be irregular or cease, unless the mechanism were out of adjustment. The horologity of the clock and mentality may be aberrant, but both conditions imply as causes illy-working mechanism.

The engineer speaks of his locomotive "working crazily," but knows that it is due to imperfection in his machine, hence its "craziness" is a symptom, and cannot exist as a condition independent of the engine.

Plaintiff's attorney asked if such views had not won for those who held them the title of "physical mediacists." I stated that such a name might apply as contradistinguishing them from metaphysicians, who held that the body had no connection with the mind.

The roof is the "physicalmedium" that shelters us from the rain, the ear and eye are the "physical media" by which we hear and see, the brain in conjunction with other bodily parts are "physical media" for mental action, and a man might as well be expected to walk without the "physical media" of legs as to think without his head.

I refused to undertake a definition of insanity as the law of relativity rendered any definition impossible. An approximation to one was contained in the statement that it was a symptom of bodily disease. The same difficulty lies in a consideration of what constitutes disease in general. This might be absence of health, and health is absence of disease. So far as that is concerned an absolute definition of anything is impossible.

The jury returned a special verdict and Judge Wylie decided in favor of the plaintiff upon technical points, having no reference to whether the mind could exist independently of the body or not.

The decision upon appeal will be one of the most important in insurance cases, as it will affect all life insurance companies.

